# Intimate Partner Violence, Depression, and Anxiety Are Associated With Higher Perceived Stress Among Both Young Men and Women in Soweto and Durban, South Africa

**DOI:** 10.3389/frph.2021.638116

**Published:** 2021-03-24

**Authors:** Tatiana E. Pakhomova, Janan Janine Dietrich, Kalysha Closson, Jenni Smit, Stefanie Hornschuh, Patricia Smith, Mags Beksinska, Thumbi Ndung'u, Mark Brockman, Glenda Gray, Angela Kaida

**Affiliations:** ^1^Faculty of Health Sciences, Simon Fraser University, Burnaby, BC, Canada; ^2^Perinatal HIV Research Unit (PHRU), Faculty of Health Sciences, School of Clinical Medicine, University of the Witwatersrand, Johannesburg, South Africa; ^3^Health Systems Research Unit, South African Medical Research Council, Cape Town, South Africa; ^4^School of Population and Public Health, University of British Columbia, Vancouver, BC, Canada; ^5^Maternal Adolescent and Child Health Research Unit, Faculty of Health Sciences, University of the Witwatersrand, Durban, South Africa; ^6^Africa Health Research Institute, Durban, South Africa; ^7^HIV Pathogenesis Programme, The Doris Duke Medical Research Institute, University of KwaZulu-Natal, Durban, South Africa; ^8^Max Planck Institute for Infection Biology, Berlin, Germany; ^9^Division of Infection and Immunity, University College London, London, United Kingdom; ^10^Office of the President, South African Medical Research Council, Cape Town, South Africa

**Keywords:** perceived stress, young people, intimate partner violence, psychosocial health, South Africa

## Abstract

**Objectives:** Psychological stress is an important determinant of health, including for mental well-being and sexual health. However, little is known about the prevalence and psychosocial and sexual health correlates of perceived stress among young people in South Africa, where elevated life-stressors are an important driver of health inequities. This study examines the association between intimate partner violence (IPV), psychosocial and sexual health, and perceived stress, by gender, among South African adolescents and young adults.

**Methods:** Using baseline survey data from AYAZAZI, a cohort study enrolling youth (16–24 years) from Durban and Soweto, we used the 10-item Perceived Stress Scale (PSS-10) to measure the degree to which an individual perceives their life situations as unpredictable, uncontrollable, and overloaded. Possible scores range between 0 and 40; higher scores indicating higher perceived stress. Crude and adjusted gender-stratified linear regression models examined associations between sexual health factors, experiences (young women) and perpetration (young men) of IPV, anxiety (APA 3-item Scale, ≥2 = probable anxiety), and depression (10-item CES-D Scale, ≥10 = probable depression) and perceived stress. Multivariable models adjusted for age, income, sexual orientation, and financial dependents.

**Results:** Of the 425 AYAZAZI participants, 60% were young women. At baseline, 71.5% were students//learners and 77.2% earned ≤ ZAR1600 per month (~$100 USD). The PSS-10 had moderate reliability (α = 0.70 for young women, 0.64 for young men). Young women reported significantly higher mean PSS scores than young men [18.3 (6.3) vs. 16.4 (6.0)]. In adjusted linear regression models, among young women experiences of IPV (β = 4.33; 95% CI: 1.9, 6.8), probable depression (β = 6.63; 95% CI: 5.2, 8.1), and probable anxiety (β = 5.2; 95% CI: 3.6, 6.8) were significantly associated with higher PSS scores. Among young men, ever perpetrating IPV (β = 2.95; 95% CI: 0.3, 5.6), probable depression (β = 6; 95% CI: 4.3, 7.6), and probable anxiety (β = 3.9; 95% CI: 2.1, 5.8) were significantly associated with higher perceived stress.

**Conclusion:** We found that probable depression, anxiety, perpetration of IPV among young men, and experiences of IPV among young women, were associated with higher perceived stress. Critical efforts are needed to address the gendered stressors of young men and women and implement services to address mental health within violence prevention efforts.

## Introduction

Stress is an important determinant of both psychological and physical well-being, playing a significant role in influencing numerous health outcomes (1, 2). Broadly defined, stress refers to the adaptive biological and psychological changes which occur in response to external demands, stimuli, or changes in the environment (3). Research on the nature of stress and its relationship to health outcomes includes complex interactions between developmental, psychological, biological, and socio-structural factors (4). While there is evidence that short-term stress can be protective (5), long-term stress response activation is linked to immune system dysregulation and changes in the pathological inflammatory response (5, 6), as well as modification of gene expression (7), playing an important role in predicting lowered health outcomes (7–12). Chronic stress has been linked to mental health conditions such as depression and anxiety (1, 13), cardiovascular disease, systemic inflammation, upper respiratory infections, and obesity (14), as well as poor sexual and reproductive health outcomes (15).

Lazarus and Folkman's theory of Stress and Coping (16) posits that stress is a dynamic process and that an individual's experience of stress is determined by how they interpret and in turn respond to or cope with presenting stressors. Perceived stress, which refers to the subjective perceptions of capacity to cope with stressful events or situations (1), is thus a commonly used measure to interpret or appraise psychological activation in response to environmental stressors (17). Rather than measuring the types or prevalence of stressful events, it examines the degree to which an individual finds their life unmanageable and unpredictable, and evaluates perceived ability to cope with specific situations or difficulties that may arise (13, 17, 18). As such, important factors influencing the relationship between stress and health are tools and mechanisms to cope with stressors, as well as self-efficacy – which refers to an individual's belief regarding their capacity to manage stressful situations (19). Coping skills, broadly categorized as behavioral and cognitive measures taken in response to stressful life events and adversities (20, 21), and include both active coping strategies, as well as maladaptive or passive/disengaged coping strategies (20, 22) which are associated with a number of health risk behaviors (21, 23). Studies examining the relationship between self-efficacy, coping, and stress have found that lowered self-efficacy (24) and limited coping resources and associated behaviors (13) are significantly correlated with high perceived stress, and influence future vulnerability via the mutually reinforcing stress-coping dynamic (16, 22, 25). The stress-generation hypothesis further suggests that exposure to elevated life stressors and negative life events plays a role in predicting future sensitivity to stress and adverse health (22). Recurring or ongoing life-stressors, which include socio-economic and family strains (26), violence (including intimate partner violence) (13, 27), as well as structural and socio-political factors (28) such as poverty (29), colonization (30), and heightened chronic stress due to gender inequity, racism, and homophobia (25, 31, 32), have been recognized as having a significant effect on poor physical and psychological health. As such, high, chronic psychological stress is an important contributor to the global disease burden, in particular for structurally marginalized communities (28) due to the high prevalence of life stressors, structural inequities, healthcare exclusion, and limited mental health and social supports (33, 34).

For young people, experiences of stress need to be contextualized within the unique vulnerability brought on by the transition into adulthood, where intersecting biological, socio-structural and psychosocial changes associated with development influence and shape health and well-being over the course of their lives (35, 36). Global data on mental health among young people has consistently found strong associations between exposure to stressors in early life, such as traumatic life events, violence, and socio-economic inequities, and adverse health outcomes in adulthood (35, 37), including depression susceptibility and increased sensitivity to later life stressors (4, 38). It is estimated that 10–20% of adolescents worldwide, aged 10–19, have mental health disorders, with a larger proportion experiencing lowered mental health symptoms which can affect well-being (39). Literature on mental health outcomes such as depression reports a consistently strong relationship with lowered sexual and reproductive health, indicating that depression can be both a risk factor for and a consequence of sexual risk behavior (40). Research has found that psychological stress among young women is associated with increased sexual risk behaviors such as unprotected sex, non-monogamous partners, lowered condom negotiation communication, relationship power imbalances and experience of dating violence (41), and decreased and inconsistent condom (20), increased number of partners as well as higher risk sexual partners (42), and incident STIs (15, 43). Furthermore, perceived stress has been found to be independently associated with a greater incidence of bacterial vaginosis (BV) (44) which has consistently been shown to increase STI acquisition risk (15).

In South Africa, a demographically young country with just under half of the population below the age of 25 (45), high socio-economic inequities are a major driver of poor health and are endemic, multidimensional, and racialized (46), and tied to the historical institutional discrimination and segregation of Apartheid and colonization (46, 47). Research from South Africa, consistently reports high rates of mental health conditions among South Africans (34, 47–51), including a high prevalence among adolescents and young adults (52, 53). Estimates of depression and anxiety disorders range from 4% (53) to 16% (54), with significantly higher rates of depressive symptoms reported among this population (55, 56), and persistent disparities in mental health outcomes are reported among young women compared with young men (52, 55, 57, 58). Research also indicates socio-economic (59) and rural-urban differences in mental health symptoms for adolescents aged 15–19 (60), with urban adolescents reporting higher probable depression compared with adolescents living in rural areas (14.6 vs. 9.4%) (60). Studies examining psychological stress have found strong associations between environmental stressors and low psychological and physical well-being in South African adolescents (61), with high levels of stress among South African university students associated with negative academic adjustment (62) and reporting depressive symptoms (63, 64). Among young South Africans lowered mental health outcomes are highly correlated with experiences of trauma (65) and violence (55, 59) including intimate partner violence (IPV), a known HIV risk factor (55, 56, 58, 66).

Given the strong empirical relationship between stress and lowered mental health outcomes (4) and the elevated presence of life stressors such as violence, including IPV, as well as poverty and gender inequity (47, 67), it is important to understand and identify factors that affect experiences of stress, as accurate epidemiological descriptions are central to formulating appropriate prevention and management of mental health for young people. Furthermore, because the young people included in our study are from communities with elevated risk for poor sexual and reproductive health outcomes (68), we were interested in examining whether sexual health is associated with perceived stress, consistent with extensive literature on mental health and sexual health outcomes (40, 56, 69).

To better understand the prevalence and correlates of perceived stress among young people in South Africa, this paper assesses the gendered influences of specific factors related to psychosocial and sexual health, inclusive of intimate partner violence (IPV), mental health, and sexual health covariates and their relationships to perceived stress, among adolescents and young adults. We hypothesize that young men and women experiencing higher perceived stress will be more likely to report engaging in sexual health risk behaviors, be more likely to report probable depression and anxiety, and intimate partner violence.

## Methods

### Study Overview and Setting

This analysis uses baseline data from an interview-administered cross-sectional survey conducted among 425 adolescents aged 16–24 years enrolled in the AYAZAZI study in South Africa. The AYAZAZI study (meaning “knowing themselves” in Zulu, one of the 11 official South African languages) was an interdisciplinary cohort study assessing the intersecting socio-behavioral, structural, and biomedical HIV risk factors among HIV-negative or HIV-status unknown young people in Soweto and Durban, South Africa. The study was guided by a youth-engagement framework, which involves the meaningful inclusion of adolescents and young adults as valued partners throughout the research process (70). The Soweto cohort was based out of the Perinatal HIV Research Unit (PHRU) located at the Chris Hani Baragwanath Hospital (2014–2017) in the City of Johannesburg Metropolitan Municipality in the Gauteng Province. The Durban cohort was based out of the Maternal, Adolescent and Child Health Research Unit (MRU) in Durban's central business district, in the eThekwini Municipality in the KwaZulu Natal Province.

Participants were eligible to participate if they lived in Soweto or Durban, were between the ages of 16 and 24 years, self-reported to be HIV negative or did not know their HIV status at enrolment, were not engaged in other HIV prevention studies at the time, and were able to provide written informed consent or assent along with parental consent if under the age of 18. Recruitment was conducted via community outreach using posters, pamphlets, and word of mouth, as well as through the PHRU HIV Counseling and Testing clinic and a public sector reproductive health clinic located near MRU. An age and gender stratified sampling approach was used for recruitment, targeting more young women (60%), to reflect gendered HIV risk in South Africa. Participants were followed for 12 months in Durban and 18 months in Soweto, with regular follow-up visits every 6-months at both sites; Durban had an additional follow-up visit at 3-months. At baseline and follow-up visits, participants completed a detailed socio-behavioral questionnaire administered by trained young multilingual peer-interviewers. Survey questionnaires included questions assessing socio-demographics, sexual and reproductive health, experiences of violence, and mental health, and were reviewed by both the PHRU's adult and adolescent prevention community advisory boards (CABs). Surveys were administered in the participant's preferred language (English, isiZulu, Sesotho) and completed online using DataFAX™ software. Additional information about the AYAZAZI study has been previously published elsewhere (70, 71).

### Measures

#### Primary Outcome: Perceived Stress

Perceived stress in this study was assessed using the 10-item version of the Perceived Stress Scale (PSS), a globally utilized psychological assessment tool used to measure an individual's self-perception of stress (17). The scale examines the degree to which an individual finds their life to be unpredictable, uncontrollable, and overloaded in the previous month (18, 72). The scale has been used widely and validated in a wide range of countries and cultural settings (29, 73, 74) including South Africa (51, 75). In addition to being used as a tool to describe perceived stress, the PSS has been utilized in studies examining the biological-psychological relationship of the stress process, including the relationship with immune function (76), and inflammatory response (2). The original scale developed by Cohen et al. in 1983 contained 14 items, with a modified 10-item version of the scale introduced in 1988 by the same authors, reporting comparatively more robust psychometric properties (72, 77). The PSS includes 10 questions asking about participants' feelings in the past 30 days, using a 5-point Likert response format (Never = 0, Almost Never = 1, Sometimes = 2, Fairly Often = 3, Very Often = 4). Items 4, 5, 7, and 8 are positively stated and thus scores are reversed. The possible scores range between 0 and 40 with higher scores indicating higher perceived stress. In this study the PSS-10 had moderate reliability, with a Cronbach alpha (α) score of 0.68 overall, 0.70 for young women, and 0.64 for young men. As stated by the original authors, the PSS is not a diagnostic tool, and thus cut-off scores are determined by the sample (78); previous work using the PSS reported perceived stress as both a categorical (13) and a continuous variable (15). We have chosen to report PSS scores as a continuous outcome variable to observe the role of IPV, mental health, and sexual health covariates on incremental increases in PSS scores.

#### Exposures of Interest

Exposures of interest explored in these analyses were selected based on our hypothesis regarding the potential associations between mental health outcomes, IPV, and sexual health factors, and higher perceived stress, informed by literature and theoretical frameworks examining the stress-health dynamic (4, 16).

##### Sexual Health

Because sexual behavior is thought to be associated with the stress-coping processes, based on theoretical and empirical evidence we chose to investigate whether those who reported engaging in certain sexual risk behaviors would also report higher perceived stress. To test our hypothesis with regard to sexual health we assessed whether condom use at last sex with all partners (inconsistent vs. consistent vs. no sex) in the previous 6 months was associated with higher PSS scores.

##### Intimate Partner Violence

Experiences of intimate partner violence, defined by the World Health Organization “any behavior within an intimate relationship that causes physical, psychological or sexual harm to those in the relationship” (79), were assessed in this study. Young women were asked if they had ever been physically hurt or threatened by a partner (yes vs. no) and young men were asked if they had every physically hurt or threatened a partner (yes vs. no); questions on experiences of IPV were inclusive of all participants whether they had or had not had consensual sex.

##### Depression

Probable depression was assessed using a 10-item Center for Epidemiologic Studies Short Depression Scale (CES-D 10) (80). Each item asked the participants about how they might have felt or behaved in the past 7 days using a 4-point Likert response format. The possible scores range between 0 and 30 with higher scores indicating a greater probability of depression; a cut-off score of ≥10 was used to identify probable depression (80).

##### Anxiety

In our study, we assessed probable anxiety using the American Psychiatric Association (APA) DSM-5 Self-Rated Level 1 Cross-Cutting Symptom Measure— Child Age, a validated self-administered clinical screening tool (81); for this study, we included only the 3-item Anxiety Scale. The anxiety domain in the DSM-5 Level 1 Cross-Cutting Symptom Measure assesses anxiety symptoms experienced over the past 2 weeks with the following three questions: “Felt nervous, anxious, or scared?”; “Not been able to stop worrying?”; “Not been able to do things you wanted to or should have done, because they made you feel nervous?.” Responses are rated on a 5-point scale (0 = none, 4 = severe), with a rating of 2 (mild) or higher indicating probable anxiety.

#### Potential Confounders

##### Demographics and Socio-Economic Status

The survey questionnaire included measures of both biological sex at birth (female vs. male vs. other) and gender; all participants identified as cisgender, meaning their perceived gender corresponds with their birth sex, and thus the binary variable gender (young men vs. young women) was used in this analysis. Other demographic characteristics included were age in years (16–17 vs. 18–21 vs. 22–24), sexual orientation [heterosexual vs. LGB (lesbian, gay, bisexual)], financial dependants (none vs. ≥1), and average monthly income categories (≤ZAR400 vs. ZAR401-1600 vs. >ZAR1600) which were adapted from a national survey (82).

#### Statistical Analysis

Descriptive statistics were used to summarize baseline characteristics of the participants. As PSS scores were normally distributed, bivariable analysis of associations between PSS scores, and psychosocial and sexual health covariates were assessed using the *t*-tests for variables with two response categories, and ANOVA for variables with more than two. Crude and adjusted gender-stratified linear regression models were used to examine the associations between a number of factors that had been previously found to be theoretically or empirically associated with perceived stress and PSS scores. We ran separate independent models assessing the associations between IPV experiences (among young women), IPV perpetration (among young men), probable depression (using the 10-item Centre for Epidemiology Depression Scale, ≥10 = probable depression), probable anxiety (APA Anxiety Measure, ≥2 = probable anxiety), and sexual health (condom use) and PSS scores. Multivariable models were adjusted for age, sexual orientation, financial dependents, and income. Variables included in the analysis were checked for multicollinearity; no multicollinearity was found among independent variables [variance inflation factor (VIF) scores < 1.42; tolerance indicators > 1.03]. Statistical analysis was performed using Statistical Analytics Software (SAS) version 9.4.

## Results

### Descriptive Baseline Characteristics

Of the 425 study participants enrolled in the study, 220 were from Soweto and 205 from Durban. The median age was 19 (IQR = 18–21), 59.5% were young women and 40.5% young men, 7% identified as LGB, with all participants identifying as cis-gender; at the time of the study 71.5% were students or in school (currently enrolled in primary school, high school, or post-secondary education) (Table 1). In comparison with young women, young men were more likely to earn ZAR1600 or more (27.9 vs. 19.4%), and in the 6 months prior to enrolment, more likely to report consistently using condoms with all partners (52.9 vs. 31.6%). More young women reported ever experiencing IPV than young men (13.9 vs. 7.1%), while young men were more likely to report ever perpetrating IPV compared with young women (12.9 vs. 4.4%). Overall probable depression was fairly high at baseline (42.8%), with young women significantly more likely than young men to report significant depressive symptoms (48.4 vs. 34.7%). Similarly, high rates of probable anxiety were reported overall (63%), with significant disparities between young women vs. young men (68.4 vs. 55.2%).

**Table 1:** Socio-demographic, sexual behavior, violence, and mental health factors of AYAZAZI participants overall and by gender (*n* = 425).

	**Overall (*n* = 425)** ***N* (%)**	**Young men (*n* = 172)** ***N* (%)**	**Young women (*n* = 253)** ***N* (%)**	***p*-value**
**Age**				
16–17	106 (24.9%)	42 (24.4%)	64 (25.3%)	0.8728
18–21	256 (60.2%)	106 (61.6%)	150 (59.3%)	
22–24	63 (14.8%)	24 (14%)	39 (15.4%)	
Sexual orientation^**^				
Heterosexual	395 (93.2%)	160 (93.6%)	235 (92.9%)	0.7850
LGB	29 (6.8%)	11 (6.4%)	18 (7.1%)	
Current student^**^				
No	121 (28.5%)	50 (29.1%)	71 (28.2%)	0.8412
Yes	303 (71.46%)	122 (70 93%)	181(71.83%)	
**Average monthly income**				
≤R400	124 (29.2%)	55 (32%)	69 (27.3%)	0.0204^*^
R401–R1600	204 (48%)	69 (40.1%)	135 (53.4%)	
≥R1601	97 (22.8%)	48 (27.9%)	49 (19.4%)	
**Financial dependents**				
None	303 (71.3%)	129 (75%0)	174 (68.8%)	0.1638
≥1	122 (28.7%)	43 (25%)	79 (31.2%)	
Ever had consensual sex^**^				
No	63 (14.9%)	17 (9.9%)	46 (18.1%)	0.0193^*^
Yes	361 (85.1%)	154 (90.1%)	207 (81.8%)	
Used a condom at last sex (6 months)^**^				
No Sex	99 (23.5%)	40 (23.5)	59 (23%)	<0.0001^*^
Consistently	170 (40.3%)	90 (52.9%)	80 (31.8%)	
Inconsistently	155 (36.6%)	40 (23.5%)	113 (44.8%)	
Ever experienced IPV^**^				
No	375 (88.9%)	158 (92.9%)	217 (86.1%)	0.0287^*^
Yes	47 (11.1%)	12 (7.1%)	35 (13.9%)	
Ever perpetrated IPV^**^				
No	390 (92.2%)	148 (87.1%)	242 (95.6%)	0.0012^*^
Yes	33 (7.8%)	22 (12.9%)	11 (4.4%)	
Probable Depression (CES-D 10 Scale)^**^				
No	239 (57.2%)	111 (65.3%)	128 (51.6%)	0.0055^*^
Yes	179 (42.8%)	59 (34.7%)	120 (48.4%)	
**Probable Anxiety (APA Scale)**				
No	157 (37%)	77 (44.8%)	80 (31.6%)	0.006
Yes	268 (63%)	95 (55.2%)	173 (68.4%)	

*IPV = Intimate partner violence; LGB = lesbian, gay, bisexual; CES-D 10 Scale = 10-item Center for Epidemiologic Studies Short Depression Scale, ≥10 indicates probable depression; APA Anxiety Scale = 3-item American Psychiatric Association Anxiety Measure, ≥2 indicates probable anxiety*;

* *p < 0.05*.

** *Some missing data*.

The mean baseline PSS score overall (*n* = 421) was 17.5, SD = 6.37. Observed scores ranged from 0 to 37 out of a possible 40, (1–37 for young women, 0–30 for young men), with higher scores indicating higher levels of perceived stress. Mean [M (SD)] PSS scores were higher among young women [18.3 (6.3) vs. 16.4 (6), *p* = 0.02], and older participants [19.3 (6.4) for ages 22–24 vs. 17.5 (6.3) for ages 18–21 vs. 16.5 (6.3) for ages 16–17, *p* = 0.02].

### Bivariable Differences in PSS Scores by Socio-Demographic, Sexual Health, and Violence Factors

Reporting depressive symptoms was associated with higher PSS scores both overall (<0.0001) as well as for both young women (<0.0001) and young men (<0.0001) (Table 2). Individuals identifying as LGB had higher mean perceived scores in comparison to those identifying as heterosexual [21.3 (7.1) vs. 17.3 (6.2), *p* = 0.001), as did participants who reported 1 or more financial dependents [19 (6.4) vs. 16.9 (6.3), *p* = 0.002], and were not students/in school at the time of the study [20 (5.9) vs. 16.5 (6.3), *p* < 0.0001]. PSS scores were also higher among those who reported inconsistent condom use at last sex within the last 6 months vs. consistent use or those reporting no sex [19.9 (6.3) vs. 17.4 (6.3) vs. 17 (6.5); *p* = 0.0312]. Young women who reported ever experiencing IPV vs. never [22.6 (5.5) vs. 17.6 (6.4); *p* < 0.0001], and young men who reported ever perpetrating IPV vs. never, also had higher PSS scores [19.3 (6.8) vs. 15.9 (5.8), *p* = 0.01].

**Table 2:** Mean (standard deviation) differences in PSS Scores by socio-demographic, sexual behavior, and mental health factors overall and stratified by gender (*n* = 421).

	**Overall (*n* = 421)**			**Young men (*n* = 172)**			**Young women (*n* = 249)**		
**Variable**	***N***	**Mean (SD)**	***p*-value**	***N***	**Mean (SD)**	***p*-value**	***N***	**Mean (SD)**	***p*-value**
**Gender**									
Young men	172	16.4 (6)	0.002^*^						
Young women	249	18.3 (6.3)							
**Age**			0.024^*^			0.037^*^			0.264
16–17	106	16.5 (6.3)		42	14.62 (6.7)		64	17.8 (5.8)	
18–21	253	17.5 (6.3)		106	16.58 (5.4)		147	18.2 (6.8)	
22–24	62	19.3 (6.4)		24	18.42 (6.4)		38	19.9 (6.4)	
Sexual Orientation^**^			0.001^*^			0.216			0.002^*^
Heterosexual	391	17.3 (6.2)		160	16.23 (5.9)		231	17.96 (6.3)	
LGB	29	21.3 (7.1)		11	18.55 (6.7)		18	22.94 (6.9)	
**Income category**			0.134			0.05^*^			0.665
<400 ZAR	124	16.82 (6.4)		55	15.22 (6.4)		69	18.1 (6.22)	
401–1,600 ZAR	201	17.45 (6.5)		69	16.12 (5.8)		132	18.2 (6.7)	
>1,600 ZAR	96	18.55 (6)		48	18.02 (5.5)		48	19.1 (6.5)	
Current Student^**^			<0.0001^*^			0.0006^*^			<0.0001^*^
No	121	20 (5.9)		50	18.78 (5.2)		71	20.9 (6.2)	
Yes	299	16.5 (6.3)		122	15.37 (6)		177	17.3 (6.4)	
**Financial Dependents**			0.002^*^			0.019^*^			0.07
None	299	16.9 (6.3)		129	15.7 (6.1)		170	17.9 (6.3)	
1 or more	122	19 (6.4)		43	18.2 (5.4)		79	19.4 (6.8)	
Used a condom at last sex (6 months)^**^			0.0312^*^			0.085			0.62
No Sex	99	17 (6.5)		40	15.6 (6.2)		59	17.9 (6.6)	
Consistently	169	16.9 (6.1)		90	15.9 (5.6)		79	18 (6.5)	
Inconsistently	150	18.7 (6.6)		40	18.2 (6.4)		110	18.9 (6.7)	
Ever experienced IPV^**^			<0.0001^*^			0.525			<0.0001^*^
No	372	17.1 (6.3)		158	16.3 (6.1)		214	17.6 (6.4)	
Yes	46	21.3 (5.8)		12	17.4 (4.8)		34	22.6 (5.5)	
Ever perpetrated IPV^**^			0.074			0.014^*^			0.465
No	386	17.4 (6.3)		148	15.9 (5.8)		238	18.3 (6.5)	
Yes	33	19.4 (6.6)		22	19.3 (6.8)		11	19.7 (6.7)	
Probable Depression (CES-D 10 Scale)^**^			<0.0001^*^			<0.0001^*^			<0.0001^*^
No	236	14.7 (5.5)		111	14.2 (5.5)		125	15.1 (5.4)	
Yes	178	21.11 (5.5)		59	20.2 (4.5)		119	21.5 (5.9)	
**Probable Anxiety (APA Scale)**									
No	155	14.3 (6)	<0.0001^*^	77	13.9 (6)	<0.0001^*^	78	14.6 (5.9)	<0.0001^*^
Yes	266	19.4 (5.8)		95	18.3 (5.2)		171	20 (6.1)	

*IPV = Intimate partner violence; LGB = lesbian, gay, bisexual; CES-D 10 Scale = 10-item Center for Epidemiologic Studies Short Depression Scale, ≥10 indicates probable depression; APA Anxiety Scale = 3-item American Psychiatric Association Anxiety Measure, ≥2 indicates probable anxiety*;

* *p < 0.05*.

** *Some missing data*.

### Factors Associated With Higher Perceived Stress

In unadjusted models, higher PSS scores were associated with probable depression, symptoms of anxiety, and IPV for both young men and young women. In an adjusted linear regression analysis (Table 3), stratified by gender and controlling for age, sexual orientation, income, and financial dependents, independent associations were identified between higher PSS scores and ever experiencing intimate partner violence (β = 4.33; 95% CI: 1.9, 6.8), probable depression (β = 6.63; 95% CI: 5.2, 8.1), and probable anxiety (β = 5.2; 95% CI: 3.6, 6.8) among young women in our study. For young men, higher perceived stress was significantly associated with ever perpetrating intimate partner violence (β = 2.95; 95% CI: 0.3, 5.6), probable depression (β = 6; 95% CI: 4.3, 7.6), and probable anxiety (β = 3.9; 95% CI: 2.1, 5.8).

**Table 3:** Factors associated with higher PSS 10 scores among study participants (*n* = 410).

	**Young women**		**Young men**	
	**Unadjusted** **β (95% CI)**	**Adjusted** **β (95% CI)^∧^**	**Unadjusted** **β (95% CI)**	**Adjusted** **β (95% CI)^∧^**
Ever experienced IPV	5 (2.7, 7.3)^*^	4.33 (1.9, 6.8)^*^	–	–
Ever perpetrated IPV	–	–	3.4 (0.7, 6)^*^	2.95 (0.3, 5.6)^*^
Inconsistent condom use at last sex (6 months)	0.9 (−1.2, 2.9)	−1.1 (−2.5, 2.3)	2.6 (−0.02, 5.2)	1.6 (−1.1, 4.3)
Probable depression	6.44 (5, 8)^*^	6.63 (5.2, 8.1)^*^	6 (4.4, 7.7)^*^	6 (4.3, 7.6)^*^
Probable anxiety	5.4 (3.8, 7.1)^*^	5.2 (3.6, 6.8)^*^	4.4 (2.7, 6.1)^*^	3.9 (2.1, 5.8)^*^

*IPV Intimate partner violence*;

∧ *Adjusted for age, income, sexual orientation, financial dependents*;

* *p < 0.05*.

## Discussion

In our study, we found higher perceived stress among young women compared with young men. In a gender stratified analysis controlling for demographic and socio-economic variables, significant associations were found between higher perceived stress and probable depression, anxiety, and IPV; young women experiencing IPV and young men who reported perpetrating IPV both reported higher perceived stress scores.

The mean PSS-10 score for our sample was 17.5 (SD = 6.4), which is on the moderate to higher end of reported PSS-10 scores for similar age groups (72, 75, 83–86). PSS scores were also higher for young women, and those aged 22–24 years compared with 16–21 year-olds, consistent with findings from other studies with similar populations (75, 83, 86–88). While the mechanisms behind gendered differences in experiences of perceived stress are not fully established, empirical data and conceptual theory point to the influence of socialization, coping styles, and types of stressors (4, 23), in addition to socio-structural factors (31), as being influencers of women's elevated risk.

Probable depression was strongly associated with higher perceived stress for AYAZAZI participants, just over 40% of whom reported depressive symptoms at baseline. Similarly, significantly high rates of anxiety symptoms were reported by participants, with just under two-thirds (63%) reporting experiencing symptoms. Young women reported higher rates of mental health symptoms compared with young men, consistent with global (35) and current South African data on mental health outcomes among adolescents and young adults (52, 53, 55, 58). Reported rates of depressive symptoms in other South African studies range from 21.1% for women and 13.6% for men aged 15–26 in rural Eastern Cape (56), to 33% in adolescents aged 14–19 in Soweto (55), and 45.3% for young women aged 18–30 living in informal settlements in the eThekwini Municipality (66). Though due to the cross-sectional nature of our analysis it is not possible to determine directionality, the positive bi-directional relationship between stress and lowered mental health outcomes, such as depression and anxiety, is well-substantiated in literature, where exposure to stressors and adverse life events contributes to both current and future episodes, as well as depression and anxiety subsequently increasing vulnerability to stress (4, 22, 89).

For young women in our cohort, experiencing IPV was correlated with higher PSS scores. The relationship between intimate partner violence and lowered mental health is well-established in the literature (90, 91), and our research adds to findings from other countries showing a link between experiences of IPV and experiences of perceived stress (13, 91), including among young women aged 14–25 (92). Previous research consistently shows high rates of IPV in South Africa, particularly in HIV-endemic communities (93), with several studies reporting elevated rates of PTSD and depression among women experiencing IPV (58, 94), and a positive relationship between IPV severity and mental health problems (94). Chronic gendered stressors such as discrimination, economic inequity, and high social stress, which are elevated in South Africa (55, 65), have been shown to contribute to mental health vulnerability for women experiencing violence by reducing coping abilities (13).

Perpetration of IPV was associated with higher perceived stress for young men in our study, and although not previously examined in the South African context, this association has been reported in a cohort of low-income fathers in the United States (95). IPV perpetration in South Africa is a pervasive issue (58, 93, 96) and its relationship to mental health is impacted by a number of socio-structural factors. A 2018 study by Gibbs et al., examining the associations between poverty, psychosocial health, gender power, and IPV found that IPV perpetration was significantly associated with a number of poor mental health indicators for young men aged 18–30 from an urban informal settlement in the KwaZulu-Natal Province, and influenced by socio-economic status and experiences of childhood trauma (58). Together with socioeconomic status, perpetration of violence is often discussed in relation to constructs of masculinity, and while this study does not directly investigate the relationship between gender norms and psychological stress, masculine gender-role stress – referring to the emotional strain men experience in response to fulfilling expectations of traditional masculinity (97), has been identified as a key predictor of lowered mental health, such as depression and anxiety, and IPV perpetration among men in South Africa (93, 97). Gender inequitable masculinities have also been associated with a number of other HIV-risk behaviors among men including having multiple sexual partners, reduced condom use, and substance use, increasing HIV risk for both men and their female partners (57, 97, 98). In South Africa, social determinants of health are historically situated and socially reproduced, and have multiple and complex interactions, linking co-occurring epidemics to act on and exacerbate health risks (47). Constructs of gender in the context of high social and economic inequities are thus important considerations in mental and sexual health research, demonstrating the multiple intersections of influence on health.

Although our study did not find a significant relationship between perceived stress and condom use in an adjusted multivariate analysis, other South African studies investigating correlates of trauma exposure and intimate partner violence have found that women experiencing violence were more likely to report depressive symptoms and sexual risk behaviors, including reduced condom negotiation skills (65) and transactional sex (56). Literature commonly reports strong and interconnected associations between stressful life experiences, socio-structural inequities, depression, experiences of violence, sexual risk factors, and HIV risk (28, 57), and there is some evidence that stress may also increase biological susceptibility to STI acquisition (15). Given that the associations between depression, IPV, and perceived stress we found were in HIV endemic communities, further research could consider some of the pathways in which perceived stress may play a role in affecting the elevated HIV risk for this group, particularly for young women and adolescent girls who are at an elevated risk for HIV acquisition due to increased structural, biological, and social vulnerabilities (99).

Despite South Africa's constitutionally guaranteed right to health care, and the elevated presence of mental health conditions in the country, there continues to be a lack of access to mental health services, including a substantial need to scale up services for young people (54). A recent evaluation of South Africa's national mental health care expenditure found a significant treatment gap (100), with access to mental health services constrained by a continuing lack of linguistically accessible and community appropriate psychiatric services, where it is reported that the majority of clinicians are not able to speak the languages of the patients with whom they work (101). Health disparities are further compounded by the historically inequitable distribution of health resource allocation, playing an important role in the current diminished capacity of South Africa's public health care system (47), and the limited availability and access of mental health services pose significant challenges to addressing high burdens of mental health conditions (50, 100). Furthermore, there is a noted need for culturally responsive mental health assessment and treatment practices and the scale-up of community-based approaches which move away from a Western psychological focus on individual-only level factors, to the consideration of broader socio-political, historical, economic, and community-level factors (50, 102) when working to address socio-structural determinants of mental health.

### Strengths and Limitations

Due to the cross-sectional nature of this analysis, no directionality or causality can be inferred from these results. The data is also population and age-specific, including individuals experiencing multiple structural inequities, so generalization to other settings should be undertaken with caution. Our measure of perceived stress (PSS-10) is not a diagnostic tool and only measures perceived stress in the last 30 days, thus it may not be a reliable predictor of long-term health outcomes. However, other longitudinal studies have used the PSS to measure chronic stress over time (103), suggesting that periods of heightened perceived stress may indicate more chronic manifestations of psychological stress. Similarly, included tools for depression (CES-D) and anxiety (APA Anxiety) measure symptoms over the past 7 days and over the past 2 weeks, respectively; as these are not diagnostic tools, they do not necessarily reflect the prevalence of these disorders in the study population. However, although mental health outcomes are complex and dynamic there is evidence that symptoms of anxiety and depression in early life are an important predictor of recurrent symptoms as well as anxiety and depression disorders in later life (4, 22, 104), with the trajectories of stress and lowered mental health symptoms having a reciprocal impact on one another over time (4). Given we have variability in assessment periods across the measures included in our study, including a lifetime measure of IPV and the variability in mental health symptoms experienced over time, we are unable to determine temporality. However, variability in assessment periods is a common feature in cross-sectional studies and such comparison is a frequently employed methodological approach in other studies (49, 56). Future work would benefit from longitudinal data analysis looking at perceived stress and associated factors over time to examine these relationships more closely. As we only included measures for decreased mental health in this analysis, the inclusion of non-deficit measures such as resiliency would be useful to better understand the relationship between stress, coping, and health.

## Conclusion

This study highlights important evidence on the gendered experiences of perceived stress, IPV, lowered mental health among adolescents and young adults in South Africa. Given these observed associations, critical efforts are needed to explore the pathways in which gendered stressors and experiences as well as perpetration of violence, play a role in exacerbating health inequities for young people in South Africa. This research underscores the importance of incorporating mental health screening and treatment into violence prevention efforts for both young men and women in order to reduce significantly high levels of stress. Understanding the community contexts of violence and mental health is crucial in order to design and implement effective, community-appropriate programming and support for young women and men.

## Data Availability Statement

The datasets presented in this article are not readily available because data cannot be shared publicly because they contain sensitive human subjects information. Researchers and trainees who meet the criteria for access to confidential data are asked to complete a Project and Data Request Form, which outlines proposed research questions and approaches, and submit this to the Corresponding Author for review. Requests to access the datasets should be directed to Angela Kaida, kangela@sfu.ca.

## Ethics Statement

This study was granted approval by the Research Ethics Boards of Simon Fraser University, Burnaby, Canada (2014s0413) and the University of the Witwatersrand, Johannesburg, South Africa [(HREC)−140707]; the University of KwaZulu-Natal Biomedical Research Ethics Committee granted reciprocity to the University of Witwatersrand HREC. The KwaZulu-Natal Department of Health granted additional approval. Written informed consent was obtained from each participant 18 years and older prior to participation at the PHRU and MRU. We obtained written assent for minors younger than 18 years, together with informed consent from their parental or legal guardian, before their participation in the study. Minors who turned 18 during their time on the study were re-consented once they reached 18 years of age. For each completed scheduled study visit, participants received ZAR150 (~CAD13.5) to compensate for travel costs and time spent at the study site.

## Author Contributions

The study was conceived and designed by AK, JD, MB, JS, TN, MB, and GG. Substantial contributions to data collection included JD, AK, SH, PS, MB, and JS. TP conducted data analysis, with guidance from AK and KC. TP, AK, and KC contributed to data interpretation. The initial manuscript was drafted by TP. All authors contributed substantively to this manuscript and writing and revising the final manuscript.

## Conflict of Interest

The authors declare that the research was conducted in the absence of any commercial or financial relationships that could be construed as a potential conflict of interest.

## References

[1] Cohen S Janicki-Deverts D Miller GE. Psychological stress and disease. JAMA. (2007) 298:1685–7. 10.1001/jama.298.14.1685 17925521

[2] Miller GE Cohen S Ritchey AK. Chronic psychological stress and the regulation of pro-inflammatory cytokines: a glucocorticoid-resistance model. Health Psychol. (2002) 21:531–41. 10.1037/0278-6133.21.6.531 12433005

[3] Garcia-Leon MA Perez-Marmol JM Gonzalez-Perez R Garcia-Rios MDC Peralta-Ramirez MI. Relationship between resilience and stress: perceived stress, stressful life events, HPA axis response during a stressful task and hair cortisol. Physiol Behav. (2019) 202:87–93. 10.1016/j.physbeh.2019.02.001 30726720

[4] Hammen C. Stress and depression. Annu Rev Clin Psychol. (2005) 1:293–319. 10.1146/annurev.clinpsy.1.102803.143938 17716090

[5] Dhabhar FS. Effects of stress on immune function: the good, the bad, and the beautiful. Immunol Res. (2014) 58:193–210. 10.1007/s12026-014-8517-0 24798553

[6] Aiello AE Simanek AM Galea S. Population levels of psychological stress, herpesvirus reactivation and HIV. AIDS Behav. (2010) 14:308–17. 10.1007/s10461-008-9358-4 18264753 PMC4156100

[7] Schiele MA Gottschalk MG Domschke K. The applied implications of epigenetics in anxiety, affective and stress-related disorders - a review and synthesis on psychosocial stress, psychotherapy and prevention. Clin Psychol Rev. (2020) 77:101830. 10.1016/j.cpr.2020.101830 32163803

[8] Belsky J Ruttle PL Boyce WT Armstrong JM Essex MJ. Early adversity, elevated stress physiology, accelerated sexual maturation, and poor health in females. Dev Psychol. (2015) 51:816–22. 10.1037/dev0000017 25915592 PMC4446150

[9] Li H Li W Wei D Chen Q Jackson T Zhang Q. Examining brain structures associated with perceived stress in a large sample of young adults via voxel-based morphometry. NeuroImage. (2014) 92:1–7. 10.1016/j.neuroimage.2014.01.044 24495811

[10] Lupien SJ McEwen BS Gunnar MR Heim C. Effects of stress throughout the lifespan on the brain, behaviour and cognition. Nat Rev Neurosci. (2009) 10:434–45. 10.1038/nrn2639 19401723

[11] Romeo RD. The impact of stress on the structure of the adolescent brain: implications for adolescent mental health. Brain Res. (2017) 1654:185–91. 10.1016/j.brainres.2016.03.021 27021951

[12] Fagundes CP Glaser R Kiecolt-Glaser JK. Stressful early life experiences and immune dysregulation across the lifespan. Brain Behav Immun. (2013) 27:8–12. 10.1016/j.bbi.2012.06.014 22771426 PMC3518756

[13] Catabay CJ Stockman JK Campbell JC Tsuyuki K. Perceived stress and mental health: the mediating roles of social support and resilience among black women exposed to sexual violence. J Affect Disord. (2019) 259:143–9. 10.1016/j.jad.2019.08.037 31445340 PMC6791774

[14] Mathur MB Epel E Kind S Desai M Parks CG Sandler DP. Perceived stress and telomere length: a systematic review, meta-analysis, and methodologic considerations for advancing the field. Brain Behav Immun. (2016) 54:158–69. 10.1016/j.bbi.2016.02.002 26853993 PMC5590630

[15] Turpin R Brotman RM Miller RS Klebanoff MA He X Slopen N. Perceived stress and incident sexually transmitted infections in a prospective cohort. Ann Epidemiol. (2019) 32:20–7. 10.1016/j.annepidem.2019.01.010 30799204 PMC6446572

[16] Lazarus RS Folkman S. Stress, appraisal, and coping. New York, NY: Springer Publishing Company (1984).

[17] Cohen S Kamarck T Mermelstein R. A global measure of perceived stress. J Health Soc Behav. (1983) 24:385. 10.2307/2136404 6668417

[18] Cohen S Tyrrell DA Smith AP. Negative life events, perceived stress, negative affect, and susceptibility to the common cold. J Pers Soc Psychol. (1993) 64:131–40. 10.1037/0022-3514.64.1.131 8421249

[19] Bandura A. Self-efficacy: toward a unifying theory of behavioral change. Psychol Rev. (1977) 84:191–215. 10.1037/0033-295X.84.2.191 847061

[20] Hulland EN Brown JL Swartzendruber AL Sales JM Rose ES Diclemente RJ. The association between stress, coping, and sexual risk behaviors over 24-months among African American female adolescents. (2015) 20:443–56. 10.1080/13548506.2014.951369 25159332 PMC4344413

[21] Folkman S Chesney MA Pollack L Phillips C. Stress, coping, and high-risk sexual behavior. Health Psychol. (1992) 11:218. 10.1037/0278-6133.11.4.218 1396489

[22] Liu RT Alloy LB. Stress generation in depression: a systematic review of the empirical literature and recommendations for future study. Clin Psychol Rev. (2010) 30:582–93. 10.1016/j.cpr.2010.04.010 20478648 PMC3049314

[23] Tavolacci MP Ladner J Grigioni S Richard L Villet H Dechelotte P. Prevalence and association of perceived stress, substance use and behavioral addictions: a cross-sectional study among university students in France, 2009–2011. BMC Public Health. (2013) 13:724. 10.1186/1471-2458-13-724 23919651 PMC3750571

[24] Lee J Kim E Wachholtz A. The effect of perceived stress on life satisfaction : The mediating effect of self-efficacy. Chongsonyonhak Yongu. (2016) 23:29–47. 10.21509/KJYS.2016.10.23.10.29 27996059 PMC5154683

[25] Meyer IH. Prejudice, social stress, and mental health in lesbian, gay, and bisexual populations: conceptual issues and research evidence. Psychol Bull. (2003) 129:674–97. 10.1037/0033-2909.129.5.674 12956539 PMC2072932

[26] Doom JR Gunnar MR. Stress physiology and developmental psychopathology: Past, present, and future. Dev Psychopathol. (2013) 25 (4pt2):1359–73. 10.1017/S0954579413000667 24342845 PMC3869040

[27] Wright EN Hanlon A Lozano A Teitelman AM. The impact of intimate partner violence, depressive symptoms, alcohol dependence, and perceived stress on 30-year cardiovascular disease risk among young adult women: a multiple mediation analysis. Prev Med. (2019) 121:47–54. 10.1016/j.ypmed.2019.01.016 30695719

[28] Mthembu JC Mabaso MLH Khan G Simbayi LC. Prevalence of psychological distress and its association with socio-demographic and HIV-risk factors in South Africa: findings of the 2012 HIV prevalence, incidence and behaviour survey. SSM Population Health. (2017) 3:658–62. 10.1016/j.ssmph.2017.07.009 29349254 PMC5769074

[29] Hjelm L Handa S de Hoop J Palermo T. Poverty and perceived stress: evidence from two unconditional cash transfer programs in Zambia. Soc Sci Med. (2017) 177:110–7. 10.1016/j.socscimed.2017.01.023 28167339 PMC6662605

[30] Benoit AC Cotnam J Raboud J Greene S Beaver K Zoccole A. Experiences of chronic stress and mental health concerns among urban Indigenous women. Arch Womens Ment Health. (2016) 19:809–23. 10.1007/s00737-016-0622-8 26961003

[31] Woods-Giscombé CL Lobel M. Race and gender matter: a multidimensional approach to conceptualizing and measuring stress in African American women. Cultur Divers Ethnic Minor Psychol. (2008) 14:173–82. 10.1037/1099-9809.14.3.173 18624581 PMC2553624

[32] Bailey ZD Krieger N Agénor M Graves J Linos N Bassett MT. Structural racism and health inequities in the USA: evidence and interventions. Lancet. (2017) 389:1453–63. 10.1016/S0140-6736(17)30569-X 28402827

[33] Saxena S Thornicroft G Knapp M Whiteford H. Resources for mental health: scarcity, inequity, and inefficiency. Lancet. (2007) 370:878–89. 10.1016/S0140-6736(07)61239-2 17804062

[34] Lund C Cois A. Simultaneous social causation and social drift: longitudinal analysis of depression and poverty in South Africa. J Affect Disord. (2018) 229:396–402. 10.1016/j.jad.2017.12.050 29331699

[35] Patel V Flisher AJ Hetrick S McGorry P. Mental health of young people: a global public-health challenge. Lancet. (2007) 369:1302–13. 10.1016/S0140-6736(07)60368-7 17434406

[36] Elgar FJ Pförtner TK Moor I De Clercq B Stevens GWJM Currie C. Socioeconomic inequalities in adolescent health 2002-2010: a time-series analysis of 34 countries participating in the health behaviour in school-aged children study. Lancet. (2015) 385:2088–95. 10.1016/S0140-6736(14)61460-4 25659283

[37] Garami J Valikhani A Parkes D Haber P Mahlberg J Misiak B. Examining perceived stress, childhood trauma and interpersonal trauma in individuals with drug addiction. Psychol Rep. (2019) 122:433–50. 10.1177/0033294118764918 29569991

[38] Bifulco A Bernazzani O Moran PM Ball C. Lifetime stressors and recurrent depression: preliminary findings of the adult life phase interview (ALPHI). Soc Psychiatry Psychiatr Epidemiol. (2000) 35:264–75. 10.1007/s001270050238 10939426

[39] World Health Organization. Adolescent Mental Health: Time for Action. Knowledge Summary: Women's, Children's and Adolescents' Health. Geneva: World Health Organization (2019). Available online at: https://www.who.int/pmnch/knowledge/publications/AMH.pdf

[40] Khan MR Kaufman JS Pence BW Gaynes BN Adimora AA Weir SS. Depression, sexually transmitted infection, and sexual risk behavior among young adults in the United States. Arch Pediatr Adoles Med. (2009) 163:644–52. 10.1001/archpediatrics.2009.95 19581548 PMC2796823

[41] DiClemente RJ Wingood GM Crosby RA Sionean C Brown LK Rothbaum B. A prospective study of psychological distress and sexual risk behavior among black adolescent females. Pediatrics. (2001) 108:e85. 10.1067/mpd.2001.117075 11694669

[42] Ethier KA Kershaw TS Lewis JB Milan S Niccolai LM Ickovics JR. Self-esteem, emotional distress and sexual behavior among adolescent females: inter-relationships and temporal effects. J Adoles Health. (2006) 38:268–74. 10.1016/j.jadohealth.2004.12.010 16488825

[43] Seth P Raiji PT DiClemente RJ Wingood GM Rose E. Psychological distress as a correlate of a biologically confirmed STI, risky sexual practices, self-efficacy and communication with male sex partners in African-American female adolescents. Psychol Health Med. (2009) 14:291–300. 10.1080/13548500902730119 19444707

[44] Nansel TR Riggs MA Yu K-F Andrews WW Schwebke JR Klebanoff MA. The association of psychosocial stress and bacterial vaginosis in a longitudinal cohort. Am J Obstetr Gynecol. (2006) 194:381–6. 10.1016/j.ajog.2005.07.047 16458633 PMC2367104

[45] Statistics South Africa. Mid-Year Population Estimates 2020. Statistical Release P0302. Pretoria: Department of Statistics South Africa (2020).

[46] Coovadia H Jewkes R Barron P Sanders D McIntyre D. The health and health system of South Africa: historical roots of current public health challenges. Lancet. (2009) 374:817–34. 10.1016/S0140-6736(09)60951-X 19709728

[47] Mayosi BM Lawn JE Van Niekerk a Bradshaw D Abdool Karim SS Coovadia HM. Health in South Africa: changes and challenges since 2009. Lancet. (2012) 380:2029–43. 10.1016/S0140-6736(12)61814-5 23201214

[48] Herman AA Stein DJ Seedat S Heeringa SG Moomal H Williams DR. The South African Stress and Health (SASH) study: 12- month and lifetime prevalence of common mental disorders. South Afr Med J. (2009) 99:339–44. 19588796 PMC3191537

[49] Smit J Myer L Middelkoop K Seedat S Wood R Bekker L-G. Mental health and sexual risk behaviours in a South African township: a community-based cross-sectional study. Public Health. (2006) 120:534–42. 10.1016/j.puhe.2006.01.009 16684549

[50] Schneider M Baron E Breuer E Docrat S Honikman S Kagee A. Integrating mental health into South Africa's health system: current status and way forward. South Afr Health Rev. (2016) 2016:153–63. 10.10520/EJC189311

[51] Hamad R Fernald LCH Karlan DS Zinman J. Social and economic correlates of depressive symptoms and perceived stress in South African adults. J Epidemiol Commun Health. (2008) 62:538–44. 10.1136/jech.2007.066191 18477753

[52] Kleintjes S Flisher AJ Fick M Railoun A Lund C Molteno C. The prevalence of mental disorders among children, adolescents and adults in the Western Cape, South Africa. South Afr Psychiatry Rev. (2006) 9:157–60. 10.4314/ajpsy.v9i3.30217

[53] Buckley J Otwombe K Joyce C Leshabane G Hornschuh S Hlongwane K. Mental health of adolescents in the era of antiretroviral therapy: is there a difference between HIV-infected and uninfected youth in South Africa? J Adoles Health. (2020) 67:76–83. 10.1016/j.jadohealth.2020.01.010 32269000

[54] Lund C Boyce G Flisher AJ Kafaar Z Dawes A. Scaling up child and adolescent mental health services in South Africa: human resource requirements and costs. J Child Psychol Psychiatry. (2009) 50:1121–30. 10.1111/j.1469-7610.2009.02078.x 19243477

[55] Barhafumwa B Dietrich J Closson K Samji H Cescon A Nkala B. High prevalence of depression symptomology among adolescents in Soweto, South Africa associated with being female and cofactors relating to HIV transmission. Vulnerable Child Youth Stud. (2016) 11:263–73. 10.1080/17450128.2016.1198854

[56] Nduna M Jewkes RK Dunkle KL Shai N Colman I. Associations between depressive symptoms, sexual behaviour and relationship characteristics: a prospective cohort study of young women and men in the Eastern Cape, South Africa. J Int AIDS Soc. (2010) 13:44. 10.1186/1758-2652-13-44 21078150 PMC2992477

[57] Jewkes RK Dunkle KL Nduna M Jama PN Puren A. Associations between childhood adversity and depression, substance abuse and HIV & HSV2 incident infections in rural South African youth. JAMA. (2010) 34:833–41. 10.1016/j.chiabu.2010.05.002 20943270 PMC2981623

[58] Gibbs A Jewkes R Willan S Washington L. Associations between poverty, mental health and substance use, gender power, and intimate partner violence amongst young (18-30) women and men in urban informal settlements in South Africa: a cross-sectional study and structural equation model. PLoS ONE. (2018) 13:e0204956. 10.1371/journal.pone.0204956 30281677 PMC6169941

[59] Stansfeld SA Rothon C Das-Munshi J Mathews C Adams A Clark C. Exposure to violence and mental health of adolescents: South African health and well-being study. BJPsych Open. (2017) 3:257–64. 10.1192/bjpo.bp.117.004861 29093828 PMC5643877

[60] Ajaero C Nzeadibe C Igboeli E. Rural-urban differences in the prevalence and predictors of depression among adolescents in South Africa. South Afr J Child Health. (2018) 12 (SPE):s71–4. 10.7196/SAJCH.2018.v12i2b.150923236959

[61] Brook DW Rubenstone E Zhang C Morojele NK Brook JS. Environmental stressors, low well-being, smoking, and alcohol use among South African adolescents. Soc Sci Med. (2011) 72:1447–53. 10.1016/j.socscimed.2011.02.041 21492977 PMC3090534

[62] Petersen Ih Louw J Dumont K. Adjustment to university and academic performance among disadvantaged students in South Africa. Educ Psychol. (2009) 29:99–115. 10.1080/01443410802521066

[63] Makhubela M. The relation between low self-esteem and depressive mood in a non-clinical sample: the role of gender and negative life events. J Psychol Afr. (2019) 29:54–9. 10.1080/14330237.2019.1568067

[64] van Zyl P Jouvert G Bowen E du Plooy F Francis C Jadhanandan S. Depression, anxiety, stress and substance use in medical students in a 5-year curriculum. Afr J Health Professions Educ. (2017) 9:67–72. 10.7196/AJHPE.2017.v9i2.705

[65] Closson K Dietrich JJ Nkala B Musuku A Cui Z Chia J. Prevalence, type, and correlates of trauma exposure among adolescent men and women in Soweto, South Africa: implications for HIV prevention. BMC Public Health. (2016) 16:1191. 10.1186/s12889-016-3832-0 27884181 PMC5123224

[66] Gibbs A Dunkle K Jewkes R. Emotional and economic intimate partner violence as key drivers of depression and suicidal ideation: A cross-sectional study among young women in informal settlements in South Africa. PLoS ONE. (2018) 13:1–18. 10.1371/journal.pone.0194885 29659595 PMC5901771

[67] Health Systems Research Unit. HERStory: An Evaluation of a South African Combination HIV Prevention Intervention for Adolescent Girls and Young Women (AGYW). Cape Town: South African Medical Research Council (2020).

[68] Beksinska ME Pillay L Milford C Smit JA. The sexual and reproductive health needs of youth in South Africa–history in context. South Afr Med J. (2014) 104:676–8. 10.7196/SAMJ.8809 25363052

[69] Gupta R Dandu M Packel L Rutherford G Leiter K Phaladze N. Depression and HIV in Botswana: a population-based study on gender-specific socioeconomic and behavioral correlates. PLoS ONE. (2010) 5:e14252. 10.1371/journal.pone.0014252 21170384 PMC2999532

[70] Closson K Dietrich JJ Beksinska M Gibbs A Hornschuh S Smith T. Measuring sexual relationship power equity among young women and young men South Africa: implications for gender-transformative programming. PLoS ONE. (2019) 14:e0221554. 10.1371/journal.pone.0221554 31553723 PMC6760831

[71] Kaida A Dietrich JJ Laher F Beksinska M Jaggernath M Bardsley M. A high burden of asymptomatic genital tract infections undermines the syndromic management approach among adolescents and young adults in South Africa: implications for HIV prevention efforts. BMC Infect Dis. (2018) 18:499. 10.1186/s12879-018-3380-6 30285705 PMC6171143

[72] Denovan A Dagnall N Dhingra K Grogan S. Evaluating the perceived stress scale among UK university students: implications for stress measurement and management. Stud High Educ. (2017) 44:120–33. 10.1080/03075079.2017.1340445

[73] Magaya L Asner-Self KK Schreiber JB. Stress and coping strategies among Zimbabwean adolescents. Br J Educ Psychol. (2005) 75:661–71. 10.1348/000709905X25508 16318684

[74] Lee E-h. Review of the psychometric evidence of the perceived stress scale. Asian Nurs Res. (2012) 6:121–7. 10.1016/j.anr.2012.08.004 25031113

[75] Makhubela M. Assessing psychological stress in South African university students: measurement validity of the perceived stress scale (PSS-10) in diverse populations. Curr Psychol. (2020) 1–8. 10.1007/s12144-020-00784-3

[76] Stowell JR Kiecolt-Glaser JK Glaser R. Perceived stress and cellular immunity: when coping counts. J Behav Med. (2001) 24:323–39. 10.1023/A:1010630801589 11523331

[77] Siqueira Reis R Ferreira Hino AA Romélio Rodriguez Añez C. Perceived stress scale: reliability and validity study in Brazil. J Health Psychol. (2010) 15:107–14. 10.1177/1359105309346343 20064889

[78] Remor E. Psychometric properties of a European Spanish version of the perceived stress scale (PSS). Spanish J Psychol. (2006) 9:86–93. 10.1017/S1138741600006004 16673626

[79] World Health Organization. Preventing Intimate Partner and Sexual Violence Against Women. Geneva: World Health Organization (2010).

[80] Zhang W O'Brien N Forrest JI Salters KA Patterson TL Montaner JS. Validating a shortened depression scale (10 item CES-D) among HIV-positive people in British Columbia, Canada. PLoS ONE. (2012) 7:e40793. 10.1371/journal.pone.0040793 22829885 PMC3400644

[81] American Psychiatric Association. DSM-5 Self-Rated Level 1 Cross-Cutting Symptom Measure— Child Age 11–17 (2013).

[82] Shisana O Labadarios D Rehle T Simbayi L Zuma K Dhansay A. The South African National Health and Nutrition Examination Survey, 2012: SANHANES-1: The Health and Nutritional Status of the Nation. Cape Town: HSRC Press (2014).

[83] Wongpakaran N Wongpakaran T. The Thai version of the PSS-10: an investigation of its psychometric properties. Biopsychosoc Med. (2010) 4:1–6. 10.1186/1751-0759-4-6 20540784 PMC2905320

[84] Kelli Hall S O 'connell White K Rickert VI Reame N Westhoff C. Influence of depressed mood and psychological stress symptoms on perceived oral contraceptive side effects and discontinuation in young minority women. Contraception. (2012) 86:518–25. 10.1016/j.contraception.2012.04.010 22673038 PMC3440521

[85] Amponsah M Owolabi HO. Perceived stress levels of fresh university students in Ghana: a case study. Br J Educ Res. (2011) 1:153–69.

[86] Örücü MÇ Demir A. Psychometric evaluation of perceived stress scale for Turkish university students. Stress Health. (2009) 25:103–9. 10.1002/smi.1218

[87] Nordin M Nordin S. Psychometric evaluation and normative data of the Swedish version of the 10-item perceived stress scale. Scand J Psychol. (2013) 54:502–7. 10.1111/sjop.12071 24118069

[88] Lesage F-X Berjot S Deschamps F. Psychometric properties of the French versions of the perceived stress scale. Int J Occup Med Environ Health. (2012) 25:178–84. 10.2478/s13382-012-0024-8 22528542

[89] Harrison C Loxton H Somhlaba NZ. Stress and coping: considering the influence of psychological strengths on the mental health of at-risk South African adolescents. Child Care Pract. (2021) 27:72–86. 10.1080/13575279.2019.1604492

[90] Lagdon S Armour C Stringer M. Adult experience of mental health outcomes as a result of intimate partner violence victimisation: a systematic review. Eur J Psychotraumatol. (2014) 5:24794. 10.3402/ejpt.v5.24794 25279103 PMC4163751

[91] Bacchus LJ Ranganathan M Watts C Devries K. Recent intimate partner violence against women and health: a systematic review and meta-analysis of cohort studies. BMJ Open. (2018) 8:e019995. 10.1136/bmjopen-2017-019995 30056376 PMC6067339

[92] Agrawal A Ickovics J Lewis JB Magriples U Kershaw TS. Postpartum intimate partner violence and health risks among young mothers in the United States: a prospective study. Maternal Child Health J. (2014) 18:1985–92. 10.1007/s10995-014-1444-9 24562504 PMC4142118

[93] Closson K Hatcher A Sikweyiya Y Washington L Mkhwanazi S Jewkes R. Gender role conflict and sexual health and relationship practices amongst young men living in urban informal settlements in South Africa. Cult Health Sex. (2019) 22:31–47. 10.1080/13691058.2019.1568578 30762491

[94] Peltzer K Pengpid S McFarlane J Banyini M. Mental health consequences of intimate partner violence in Vhembe district, South Africa. Gen Hosp Psychiatry. (2013) 35:545–50. 10.1016/j.genhosppsych.2013.04.001 23643034

[95] Gordon DM Moore KE Vincent W Iwamoto DK Campbell C Hunter BA. Intimate partner violence among low-income fathers: testing a stress-coping model. J Interpers Violence. (2017) 36:1634–59. 10.1177/0886260517736878 29295001 PMC6433533

[96] Jewkes R Dunkle K Koss MP Levin JB Nduna M Jama N. Rape perpetration by young, rural South African men: prevalence, patterns and risk factors. Soc Sci Med. (2006) 63:2949–61. 10.1016/j.socscimed.2006.07.027 16962222

[97] Gottert A Barrington C McNaughton-Reyes Luz H Maman S MacPhail C Lippmanh SA. Gender norms, gender role conflict/stress and HIV risk behaviors among men in Mpumalanga, South Africa. AIDS Behav. (2018) 22:1858–69. 10.1007/s10461-017-1706-9 28161801 PMC6440537

[98] Jewkes R Sikweyiya Y Morrell R Dunkle K. The relationship between intimate partner violence, rape and HIV amongst South African men: a cross-sectional study. PLoS ONE. (2011) 6:e24256. 10.1371/journal.pone.0024256 21935392 PMC3173408

[99] South African National AIDS Council (SANAC). Let Our Actions Count: South Africa's National Strategic Plan for HIV, TB and STIs 2017–2022. Pretoria: South African National AIDS Council (2017). Available online at: https://www.gov.za/sites/default/files/gcis_document/201705/nsp-hiv-tb-stia.pdf

[100] Docrat S Besada D Cleary S Daviaud E Lund C. Mental health system costs, resources and constraints in South Africa: a national survey. Health Policy Plan. (2019) 34:706–19. 10.1093/heapol/czz085 31544948 PMC6880339

[101] Swartz L Kilian S Twesigye J Attah D Chiliza B. Language, culture, and task shifting–an emerging challenge for global mental health. Global Health Act. (2014) 7:23433. 10.3402/gha.v7.23433 24581319 PMC3938800

[102] Laher S Cockcroft K. Moving from culturally biased to culturally responsive assessment practices in low-resource, multicultural settings. Profess Psychol Res Pract. (2017) 48:115. 10.1037/pro0000102

[103] Gianaros PJ Jennings JR Sheu LK Greer PJ Kuller LH Matthews KA. Prospective reports of chronic life stress predict decreased grey matter volume in the hippocampus. Neuroimage. (2007) 35:795–803. 10.1016/j.neuroimage.2006.10.045 17275340 PMC1868546

[104] Felton JW Banducci AN Shadur JM Stadnik R MacPherson L Lejuez CW. The developmental trajectory of perceived stress mediates the relations between distress tolerance and internalizing symptoms among youth. Dev Psychopathol. (2017) 29:1391–401. 10.1017/S0954579417000335 28318473 PMC6360527

